# Ferritin light chain gene mutations in two Brazilian families with hereditary hyperferritinemia-cataract syndrome

**DOI:** 10.1590/S1679-45082017RC4006

**Published:** 2017

**Authors:** Roberta Cardoso Petroni, Susana Elaine Alves da Rosa, Flavia Pereira de Carvalho, Rúbia Anita Ferraz Santana, Joyce Esteves Hyppolito, Claudia Mac Donald Bley Nascimento, Nelson Hamerschlak, Paulo Vidal Campregher

**Affiliations:** 1 Hospital Israelita Albert Einstein, São Paulo, SP, Brazil.

**Keywords:** Ferritins, Cataract, Point mutation, Genetics, Heredity, Case reports, Ferritinas, Catarata, Mutação puntual, Genética, Hereditariedade, Relatos de casos

## Abstract

Hereditary hyperferritinemia-cataract syndrome is an autosomal dominant genetic disorder associated with mutations in the 5’UTR region of the ferritin light chain gene. These mutations cause the ferritin levels to increase even in the absence of iron overload. Patients also develop bilateral cataract early due to accumulation of ferritin in the lens, and many are misdiagnosed as having hemochromatosis and thus not properly treated. The first cases were described in 1995 and several mutations have already been identified. However, this syndrome is still a poorly understood. We report two cases of unrelated Brazilian families with clinical suspicion of the syndrome, which were treated in our department. For the definitive diagnosis, the affected patients, their parents and siblings were submitted to Sanger sequencing of the 5’UTR region for detection of the ferritin light gene mutation. Single nucleotide polymorphism-like mutations were found in the affected patients, previously described. The test assisted in making the accurate diagnosis of the disease, and its description is important so that the test can be incorporated into clinical practice.

## RESUMO

A síndrome hereditária hiperferritinemia-catarata é uma doença genética autossômica dominante associada a mutações na região 5’UTR do gene da cadeia leve da ferritina. Estas mutações elevam os níveis de ferritina, mesmo na ausência de sobrecarga de ferro. Os pacientes também desenvolvem catarata bilateral precocemente, devido ao acúmulo de ferritina no cristalino, e muitos são erroneamente diagnosticados como portadores de hemocromatose, sendo tratados de maneira inadequada. Os primeiros casos foram descritos em 1995, e diversas mutações já foram identificadas. Entretanto, essa síndrome ainda é pouco conhecida. Relatamos dois casos de famílias brasileiras, não relacionadas, com suspeita clínica da síndrome, que foram atendidas em nosso serviço. Para o diagnóstico definitivo, os pacientes afetados, seus pais e irmãos foram submetidos à pesquisa de mutação do gene ferritina, por sequenciamento de Sanger da região 5’UTR. Foram encontradas mutações do tipo polimorfismo de nucleotídeo único nos pacientes afetados, já descritas anteriormente. O teste auxiliou no diagnóstico preciso da doença e é importante ser divulgado, para ser incorporado na prática clínica.

## INTRODUCTION

Ferritin is a protein composed of two fundamental subunits (light and heavy chains) and it is responsible for the storage of iron within cells. Under normal conditions, serum ferritin concentration is used in the evaluation of iron stores and also as an acute phase protein marker. Hyperferritinemia-cataract syndrome (HCS) (OMIM 600886) is an autosomal dominant disease associated with mutations in the 5’UTR region of the ferritin light chain (FTL) gene.^(1,2)^ The FTL gene, located in chromosome 19q13, contains four exons and encodes the light subunit of the ferritin protein. The FTL 5’UTR region is also known as an iron-responsive element, and together with iron-regulating proteins, participates in the mechanism of regulation of iron concentration in cells.^(3)^

When the intracellular iron concentration is low, the iron-regulating protein binds to a specific sequence in the iron-responsive element, which folds forming a loop, preventing translation of the messenger RNA. When iron is available in the cell, it binds to the iron-regulating protein, inducing its dissociation from the iron-responsive element and allowing the translation of the messenger RNA to form the ferritin light chain.

Some specific mutations in the iron-responsive element prevent it to bind to the iron-regulating protein, leading to a continuous synthesis of the FTL gene and consequently to high levels of ferritin in the absence of iron overload.^(3)^

The first case reports of this syndrome were published in 1995 by two groups, in Italy and France.^(4,5)^ Several mutations have already been identified in families from different countries,^(3)^ but this is still a disease scarcely known.

Patients with this syndrome often develop early bilateral cataract due to accumulation of ferritin in the lens. The most frequent laboratory findings in these patients are elevated ferritin, and normal levels of serum iron and transferrin saturation.^(6)^ However, many of these patients are misdiagnosed as having hemochromatosis and are not managed appropriately, often undergoing unnecessary invasive procedures.^(3)^ Thus the cases of hyperferritinemia associated with normal serum iron levels, normal transferrin saturation, and cataract should be investigated for the hereditary HCS by screening for mutations in the 5’UTR region of the FTL gene.

We herein report two cases of Brazilian families who were seen at our department with suspected HCS. The diagnoses were confirmed by finding mutations in the 5’UTR region of the FTL gene.

## CASE REPORT

### Case 1

A caucasian, 4-year-old male patient, came for an appointment due to fatigue. Anemia was investigated. Laboratory tests showed hemoglobin levels of 13g/dL and ferritin of 1,300ng/mL. The ferritin test was repeated on several occasions and in several laboratories, confirming the result.

The investigation of a mutation in the HFE gene for hemochromatosis found the p.C282Y mutation in heterozygosity. The result of magnetic resonance imaging to investigate hepatic iron overload was normal. The serology for hepatitis was negative.

The patient’s father presented congenital cataract and was operated on. Due to the family history, the patient was often submitted to routine ophthalmologic examinations. He had indeed received a diagnosis of cataract a few months before. The presumptive diagnosis of HCS was made, and a test to detect mutation in the FTL gene was requested to confirm diagnosis.

**Figure 1:**
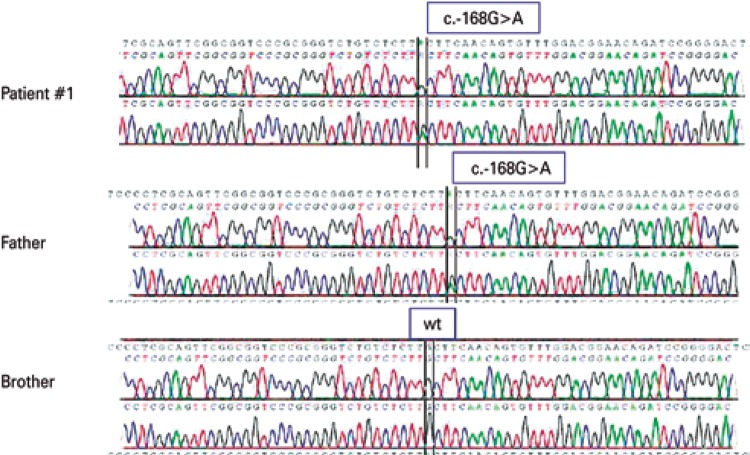
Electropherogram of the three individuals tested in the first case, evidencing the exchange of the base at position c.-168 from G to A in the affected individuals and the wild genotype in the healthy individual

We performed Sanger sequencing of the 5’UTR region of the FTL gene (Chart 1). The c.-168G>A mutation, first described in 1997, was found in this case (Figure 1).^(7)^ The mutation screening was performed on the father and on the affected child’s sibling. While the father had the same mutation, the brother presented the wild type FTL.

**Chart 1:** Primers used for amplification and sequencing of the 5’UTR region of the ferritin light gene

FTL_e01f: 5'- CTATGTGCTCCGGATTGGTC -3'
FTL_e02r: 5'- CCGAACTCAATCTCCCAGAA -3'

FTL: ferritin light.

### Case 2

**Figure 2:**
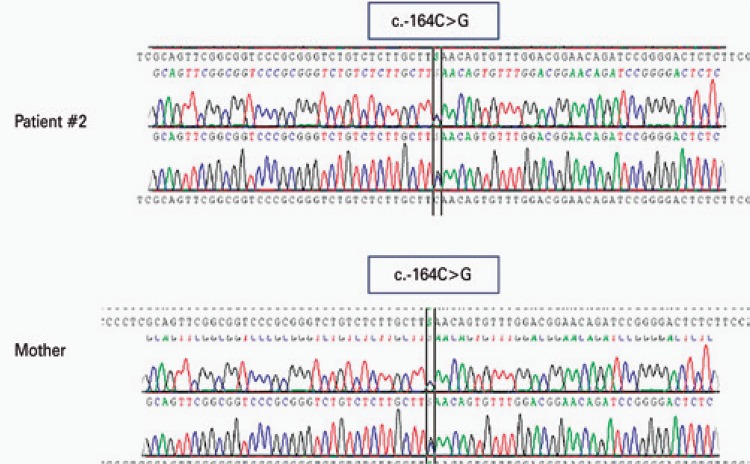
Electropherogram of the second patient and his mother, both affected, evidencing the same mutation in the c.-164 region of the ferritin light gene, in which the substitution of a C by G occurs

A 7-year-old white male patient was taken to the hematologist’s office due to increased serum levels of ferritin (1,019ng/mL) and hemoglobin of 12g/dL as incidental findings (the patient was asymptomatic). Maternal relatives of the child had a family history of congenital cataract. Transferrin saturation was 22%. The test for hemochromatosis mutation was negative. The patient was also negative for hepatitis. The sequencing of the 5’UTR region of the FTL gene demonstrated the presence of the c.-164C>G mutation (Figure 2), first described in 2003.^(2)^ The patient was referred for ophthalmologic evaluation, when he was diagnosed as congenital cataract. The boy is still being followed-up.

Mutation screening was also performed on the mother, who had a normal blood count, ferritin of 1,280ng/mL. She was operated on for cataract at 40 years. The c.-164C>G mutation in the FTL gene was also found in the mother.

## DISCUSSION

Hyperferritinemia-cataract syndrome is a rare disease caused by mutations in the 5’UTR region of the FTL gene. While the prevalence of this syndrome has been estimated to be approximately 1/200,000 in Australia,^(8)^ it is unknown in Brazil. Its clinical manifestations are congenital cataract and persistent hyperferritinemia, not associated with iron overload. Several frequent conditions are associated with congenital cataract,^(5)^ and because of that, the search for mutation in the FTL gene is rarely included in the evaluation of congenital cataract, and most patients affected by this syndrome end up being evaluated by hyperferritinemia. As mutations in the HFE gene associated with hemochromatosis are frequent in our population,^(7,9-11)^ this is often the main presumptive diagnosis for these patients. The HFE gene is located on 6p22.2 and encodes a membrane protein that is thought to control iron absorption, by regulating the interaction of the transferrin receptor with transferrin. Mutations in this gene cause hereditary hemochromatosis, a recessive genetic disorder, characterized by increased iron absorption and variable degrees of organ toxicity related to iron overload. It is noteworthy mentioning that co-occurrence of mutations in the FTL and HFE gene is possible,^(12,13)^ and can lead to unnecessary diagnoses and therapeutic phlebotomies.^(13)^

We described here two cases of unrelated patients with HCS caused by different mutations. The c.-168G>A mutation, also known as Pavia-1, has been described in families of Italian, Indian, German and Dutch descent, while c.-164C>G was first described in 2003, in a family of Italian origin.^(4,5)^ As far as we know, this is the third mutation described in Brazilian families.^(9,12)^ The father of the first patient also harbored a compound heterozygous mutation in the HFE gene, p.C282Y and p.H63D. Although this compound heterozygous genotype is not normally associated with hemochromatosis, it can cause iron overload when associated with other comorbidities.^(14)^ Therefore it is essential for all clinicians dealing with iron overload to have the diagnosis of HCS in mind in order to avoid unnecessary therapeutic phlebotomies.^(13)^

## CONCLUSION

In conclusion, we have documented two Brazilian families harboring distinct ferritin light mutations associated with hyperferritinemia-cataract syndrome. Reports such as this are needed, since the prevalence of this condition in our country is unknown and the correct diagnosis is essential to avoid unnecessary interventions.
